# Contrast enhanced mammography in breast cancer surveillance

**DOI:** 10.1007/s10549-023-06916-0

**Published:** 2023-03-25

**Authors:** Kenneth Elder, Julia Matheson, Carolyn Nickson, Georgia Box, Jennifer Ellis, Arlene Mou, Clair Shadbolt, Allan Park, Jia Tay, Allison Rose, Gregory Bruce Mann

**Affiliations:** 1 The Royal Melbourne Hospital, Grattan Street, Parkville, Melbourne, 3101 Australia; 2 Daffodil Centre, The University of Sydney, a joint venture with Cancer Council New South Wales, Sydney, Australia; 3 Melbourne School of Population and Global Health, The University of Melbourne, Parkville, Australia; 4 The Royal Women’s Hospital, Flemington Road, Parkville, Melbourne, Australia; 5 Department of Surgery, The University of Melbourne, Parkville, Australia

**Keywords:** Breast cancer, Surveillance, Mammography, Contrast, CEM

## Abstract

**Purpose:**

Mammography (MG) is the standard imaging in surveillance of women with a personal history of breast cancer or DCIS (PHBC), supplemented with ultrasound. Contrast Enhanced Mammography (CEM) has higher sensitivity than MG and US. We report the performance of CEM compared with MG ± US.

**Methods:**

A retrospective study of patients undergoing their first surveillance CEM in an Australian hospital setting between June 2006 and October 2020. Cases where a patient was recalled for assessment were identified, recording radiology, pathology and treatment details. Blinded re-reading of recalled cases was performed to determine the contribution of contrast. Use of surveillance US across the board was assessed for the period.

**Results:**

73/1191 (6.1%) patients were recalled. 35 (48%) were true positives (TP), with 26 invasive cancers and 9 cases of DCIS, while 38 (52%) were false positive (FP) with a positive predictive value (PPV) 47.9%. 32/73 were recalled due to MG findings, while 41/73 were only recalled due to Contrast. 14/73 had ‘minimal signs’ with a lesion identifiable on MG with knowledge of the contrast finding, while 27/73 were visible only with contrast. 41% (17/41) recalled due to contrast were TP. Contrast-only TPs were found with low and high mammographic density (MD). Screening breast US reduced by 55% in the year after CEM was implemented.

**Conclusion:**

Compared to MG, CEM as a single surveillance modality for those with PHBC has higher sensitivity and comparable specificity, identifying additional malignant lesions that are clinically significant. Investigation of interval cancer and subsequent round outcomes is warranted.

## Introduction

Women with a personal history of breast cancer (PHBC) have an increased incidence of subsequent in-breast malignancy, either in the form of local recurrences after breast conserving therapy or contralateral breast cancer [1]. Early detection of these events is associated with improved survival [2]. Breast cancers in patients with PHBC are twice as likely to be stage IIB or greater or to be node positive when compared with screen-detected cancers in the general population, which can translate into poorer outcomes [1, 3]. For this reason, breast imaging is a critical part of follow-up care. Clinical guidelines for women with PHBC universally recommend annual imaging, even where national guidelines for population screening may recommend 2- or 3-yearly imaging [4].

Mammography (MG) has been the foundation for surveillance after breast cancer for many years. Full field digital MG (2DMG) initially replaced analogue imaging, and more recently, digital breast tomosynthesis (3DMG) has become widespread, due to its improved cancer detection rate compared with 2DMG [5–7].

In recognition of false negatives if relying on MG alone, supplemental ultrasound (US) is often used, with reports of identification of additional lesions in up to 5.3 per 1000 patients with an elevated risk of breast cancer and high mammographic density (MD) [7–10]. However, the sensitivity of supplemental US is likely lower for women with PHBC [11]. Adjunctive screening US has low specificity, with many additional benign lesions being identified and investigated, of detriment to both the patient via heightened anxiety and to the health system via increased costs [9, 10, 12–14].

MRI has also been used in surveillance, usually in association with MG, and provides increased rates of cancer detection and lower interval cancer (IC) rates [15]. MRI is considered to be particularly helpful in cases with high MD, which is itself associated not only with increased risk of cancer or local recurrence, but also with reduced sensitivity of MG [16]. However, MRI is a more expensive test, and its limited access and capacity in Australia are barriers to its widespread use for routine surveillance.

Contrast enhanced mammography (CEM) may have a role in surveillance. The equipment and procedures are very similar to conventional MG, and it uses standard iodinated intravenous contrast. Allergic reactions are uncommon (less than 1%) and usually mild and self-limiting [17]. The radiation dose is estimated to be 20–80% higher than conventional MG but still within quality assurance guidelines [18]. In both screening and diagnostic settings, CEM has been shown to offer increased sensitivity compared with 2D/3DMG plus US, and potentially better specificity than MRI [19–21]. Published results vary on whether CEM provides comparable cancer detection to MRI [19–21]. Reports of CEM for routine surveillance after PHBC are limited, but it is potentially an attractive technology in this setting [22–25].

CEM was introduced at The Royal Melbourne Hospital, an academic hospital in Melbourne, Australia, in 2015. From late 2018 we introduced a policy that CEM alone would be the default method for all suitable, consenting patients undergoing surveillance due to PHBC, on the basis that it was expected to equal or improve on the prior policy of 2D/3DMG plus selective US. This report describes outcomes of the first round of surveillance CEM.

## Material and methods

All women with PHBC having surveillance CEM between June 2016 and October 2020 were identified. CEM includes recombined contrast images, and 2D and 3D images without contrast, all in a single acquisition. Those with known contrast allergies were excluded. Those over 65 or with a history of renal disease or diabetes had eGFR assessed and if eGFR was less than 30 ml/min CEM was not offered. Those who declined had 2D/3DMG and selective US.

CEM was performed using a Hologic 3 Dimensions unit (Hologic, Danbury, Connecticut, USA). Patients were given 100mls of Omnipaque™ 350 (Iohexol; GE Healthcare) intravenously, through a 20-gauge cannula using a power injector, at a rate of 3 mL/sec. and once the contrast injection was completed, the patient was positioned for her MG (after 2 min of injection). Mammographic imaging was usually performed in “Combo Mode” with almost simultaneous low (26–30 kVp) energy, high (45–49 kVp) energy and tomographic images interleaved. This provides 2D images, 3D images and recombined contrast-enhanced images subsequently used in analyses. Mediolateral oblique and craniocaudal views of each breast were obtained in all patients; the side of the previous cancer was usually imaged first. The imaging window was from 2 to 8 min. The low-energy images were interpreted as the 2D MG component. Postprocessing with a recombination algorithm provided an iodine (C +) image that highlighted the areas of contrast enhancement. The low keV, tomographic, and the iodine(C +) images were co-registerable, and stored in PACS for reporting. Contrast enhancement appearing above that of background was reported as requiring recall for further assessment.

In this context, recall was defined “as any intervention instigated on clinical or radiological grounds arising from a surveillance episode”. The interventions could include targeted US, problem solving MRI, early review CEM, percutaneous image guided biopsy and excisional biopsy. Since contrast guided biopsy was not available in Australia at the time of this study, biopsy was directed by stereotactic MG or US where the lesion was identified with certainty, and by MR for contrast-only lesions. A clip was deployed after all biopsies and a mammogram (CC, and MLO views with additional clip profiles used for cases of multiple lesions to allow co-registration with original CEM), sometimes with contrast, was performed to confirm concordance. A True Positive (TP) recall was defined as a recall where the final histopathology was either DCIS or invasive cancer. A False Positive (FP) recall was any other case of recall.

Data was extracted from the Radiology Department Information System, Hospital Medical Records and the Breast Service database. This included patient demographics, details of the index invasive cancer or DCIS, automated MD measurement (VOLPARA Health Technologies) and lesion classifications including lesion type and BI-RADS score. The degree of background parenchymal enhancement (BPE) on CEM was graded adapting the BI-RADS classification of BPE for MRI. All biopsies resulting from surveillance imaging were identified and histopathology and treatment was detailed.

Reporting of the images was performed by at least one breast specialised radiologist. Most were very experienced (7–40 years) in reporting breast imaging. Many of the studies were double reported by consensus as a means of increasing the experience of more of the breast radiologist group.

Since a single CEM was used as a replacement for 2D/3DMG with selective whole breast US, the reduction of supplemental US screening was also assessed. Data on utilization of supplemental breast US screening was collected for the year prior to implementation of CEM and compared with that for the following year.

Because the CEM was performed for the most part as a single acquisition, and interpreted as a single examination, the contribution of the 2D/3DMG and C + to the recall outcomes were evaluated separately. Blinded re-reading of the 2D/3DMG alone was performed by 4 experienced breast radiologists (with a range of 7–40 years’ experience) and the findings were subsequently classified as negative, minimal signs, or suspicious. “Minimal signs” were defined as cases where prospective reading of the 2D/3DMG was negative, but re-reading with the knowledge of the results of the contrast component enabled identification of the lesion of interest in non-enhanced images. Findings classified as “negative” or “minimal signs” were analysed as “C + directed”, while those with “suspicious” findings on 2D/3DMG and C + were classified as “2D/3DMG & C + ”.

Outcomes were described in terms of recalls to assessment and biopsy rates according to MD, the distribution of MD and BPE among surveilled women, results of cases recalled for further assessment according to the contribution of MG and CEM, and the profile of patients and cancers according to the contribution of CEM imaging. Data was tabulated in aggregate form with statistical tests applied using Stata 15.0 [26].

This project was approved as a Quality Assurance project by the Melbourne Health Research and Ethics Committee, QA2019129.

## Results

1191 women with a personal history of DCIS or invasive breast cancer underwent an initial surveillance. The characteristics of this patient group are outlined in Table 1. Most had previously been treated for invasive cancer and most underwent breast conserving surgery (BCS).

**Table 1:** Description of all patients with PHBC receiving CEM during the study period

N	1,191
Age (mean (SD), median (range))	59 .3 (10.0), 59 (28–92)
Time since surgery (months) (median, range)	36 (2–235)
Index pathology (N (%))	
DCIS	189 (16%)
Invasive cancer	985 (83%)
Missing	17 (1%)
Index surgery (N (%))	
BCS	969 (81%)
Mastectomy	222 (19%)

MD and BPE across the study group is summarised in Table 2. Background parenchyma tended to be more enhanced with increasing breast MD (Table 2), although correlation was modest (Spearman's *r*_*s*_ = 0.28, *p* < 0.0001). Overall, while moderate or marked BPE was infrequent (7% of all patients), 78% of moderate or marked BPE arose in the 44% of women with Category C/D MD (OR 4.5 (95% CI 2.6–7.8), *p* < 0.001), and the proportion women with minimal or mild BPE decreased from 99% for MD category A to 77% for MD category D.

**Table 2:** The distribution of BPE and breast density in the surveilled cohort

Background Parenchymal Enhancement	MD BIRADS				Total
	A	B	C	D	
Minimal	51 (74%)	403 (68%)	208 (48%)	25 (27%)	687 (58%)
Mild	17 (25%)	174 (29%)	186 (43%)	46 (50%)	423 (36%)
Moderate	1 (1%)	15 (3%)	37 (8%)	15 (18%)	68 (6%)
Marked	0 (0%)	2 (0%)	6 (1%)	5 (6%)	13 (1%)
Total	68 (100%)	582 (100%)	429 (100%)	90 (100%)	1,191 (100%)

**Fig. 1:**
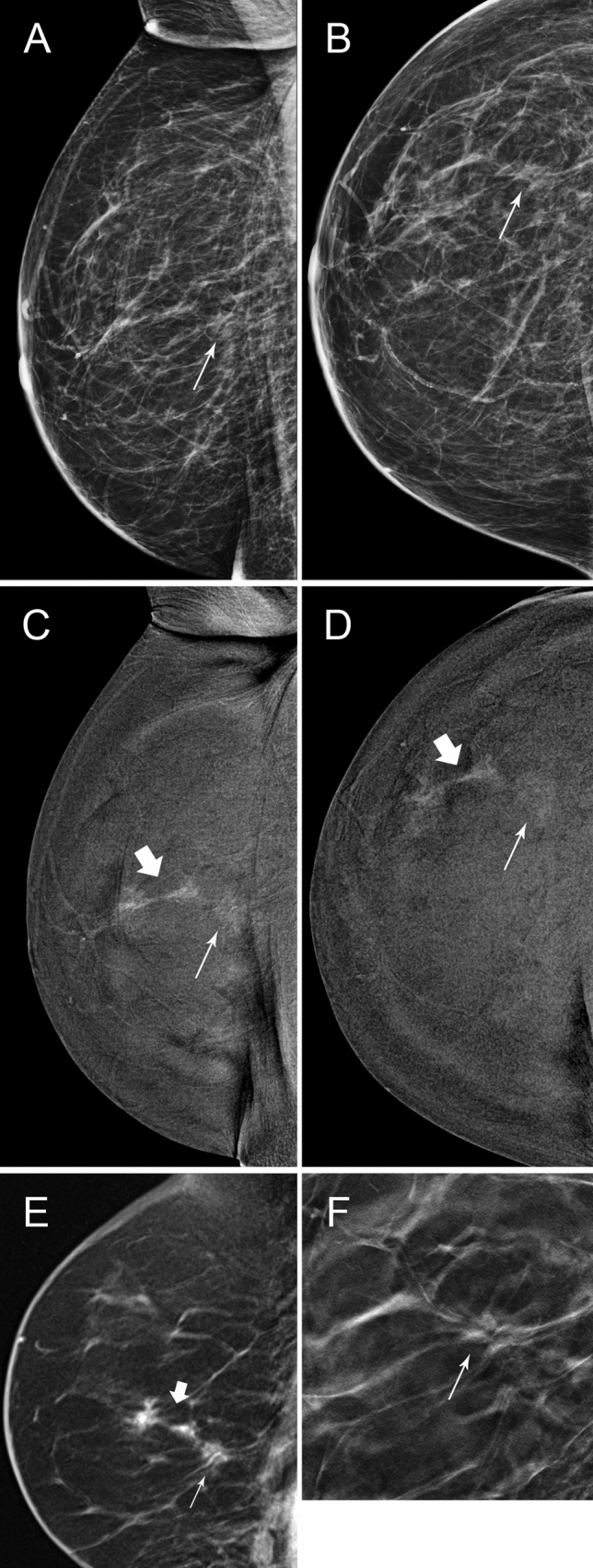
**A** and **B**, 72 year-old patient, 2D 2019 surveillance images, MD BIRADS B. She had right wide local excision 2009 for a small Gd 3 invasive node-positive cancer. Thin arrow shows a small spiculated density thought to be part of the scar, stable on tomography. F is the CC tomographic image of this density(thin arrow). **C** and **D** are C + images from the same surveillance study showing faint enhancement (thin arrow) in the spiculated density, but a further 35 mm of spiculated enhancement anteriorly (thick arrow), not seen on 2D, 3D or targeted ultrasound. E is a sagittal contrast enhanced MR image (thin MIP) at the time of MR guided biopsy showing excellent correlation of CEM & MR images. Right mastectomy showed invasive carcinoma NST, Grade 3 with 2 tumours 22 &14 mm, triple negative, Ki67 20%. SNB negative. This was classified as a minimal signs case on 2D/3D after re-reading

**Fig. 2:**
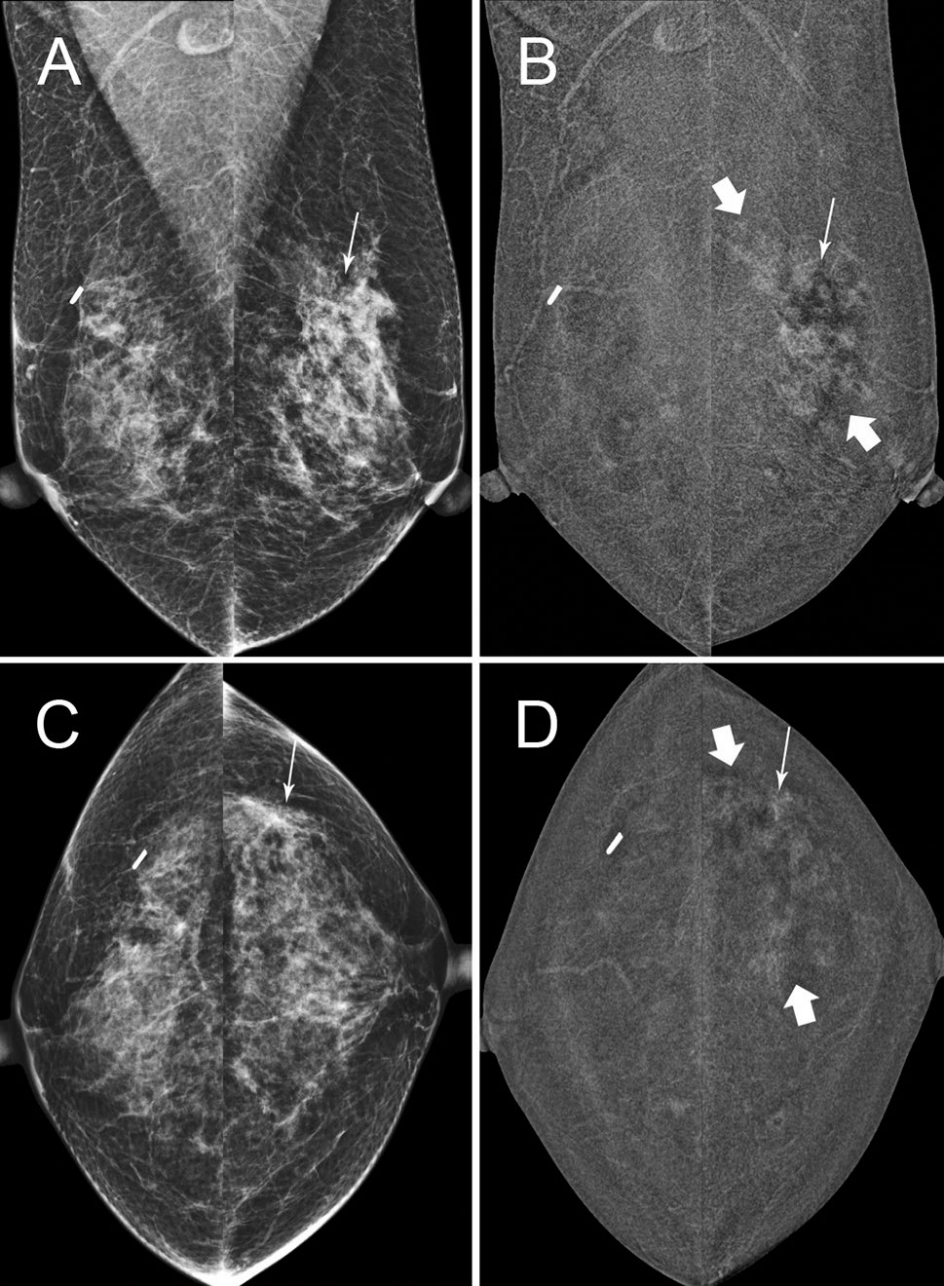
**A** and **C**, 53 year-old patient, 2D 2019 surveillance images, MD BIRADS C. She had right wide local excision 2015 for 20 mm Intermediate grade DCIS and excision of papilloma with atypia on the left in the upper outer quadrant at the same time. Thin arrow on left is site of scar, stable on tomography. No other abnormalities on 2D,3D or ultrasound on the left and no calcifications. **B** and **D** are C + images from the same surveillance study showing extensive non-mass enhancement (thick arrows) throughout the left upper outer quadrant and mild BPE bilaterally. Stereotactic biopsy targeted to the enhancing scar (thin arrow) in the left upper outer quadrant was performed. Mastectomy showed 9 cm intermediate grade DCIS without invasion. SNB negative. This is an example of a contrast-only lesion

**Fig. 3:**
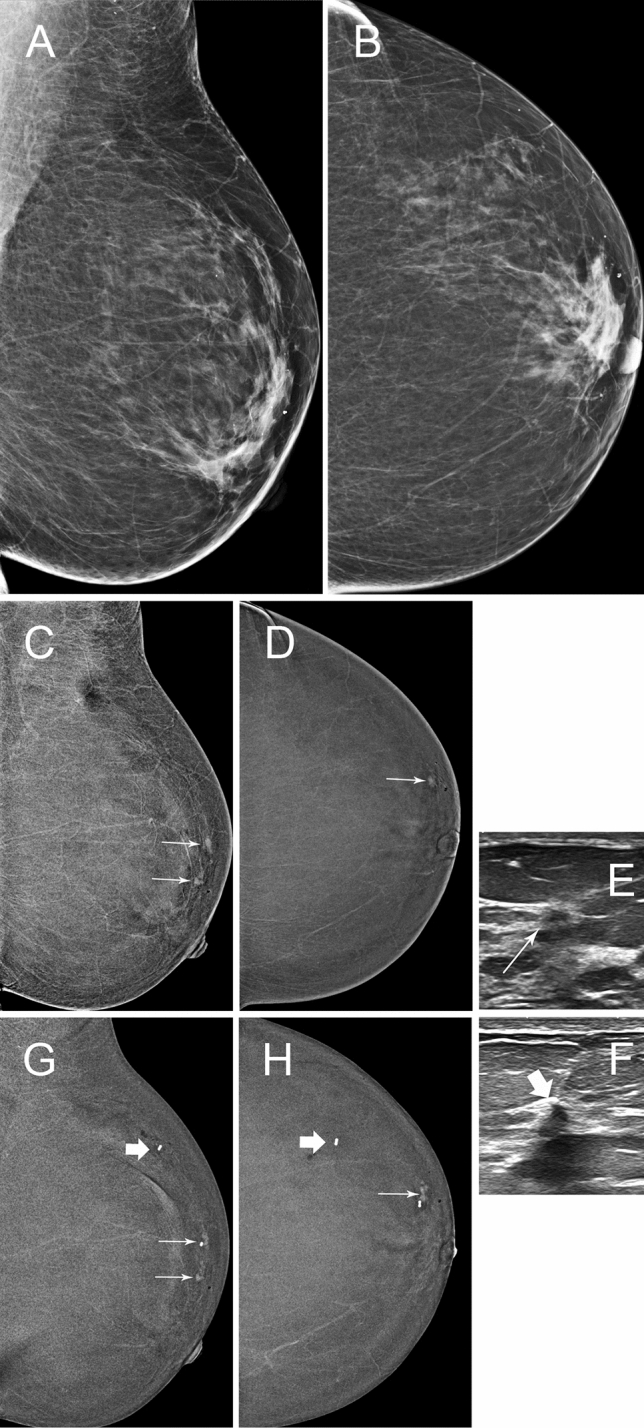
**A** and **B**, 45 year-old patient, 2D 2019 surveillance images, MD BIRADS B. She had right wide local excision 2017 for 12 mm invasive carcinoma NST with negative SNB. There were no abnormalities on 2D/3D. **C** and **D** are the C + images from the same surveillance study and show two small foci of enhancement (thin arrows) just superolateral to the nipple. Targeted ultrasound, **E** and **F**, showed two subtle hypoechoic lesions at 12.30 o’clock 2 cm(E, thin arrow) from nipple and 12.30 o’clock 5 cm(F thick arrow) from nipple, thought to correlate. US guided biopsies were performed and tissue markers were placed. **G** and **H**, post biopsy CEM shows that only one of the 2 lesions was correctly localised (thin arrow). It showed invasive carcinoma. The other biopsy contained only normal breast tissue on histopathology(thick arrow). Left wide local excision contained two foci of invasive carcinoma grade1, 4 mm & 1 mm and 25 mm of high grade DCIS. Post biopsy CEM is an excellent method of confirming that the correct lesion has been sampled. Ultrasound can present several “likely” lesions especially when the contrast lesion is small. In these instances MR or CEM guided biopsy, if available is more reliable and expeditious

Surveillance imaging led to findings warranting further investigation and/or biopsy in 73 patients (recall 6.1%). 35 (48%) were true positives (TP), with 26 invasive cancers and 9 cases of DCIS, while 38 (52%) were false positives (FP) giving a positive predictive value of recall (PPV) 47.9%. (Table 3), with no significant difference in recall rates according to MD (5.7% for BIRADS categories A/B versus 6.6% for categories C/D, χ^2^ = 0.4, *p* = 0.52). Seven cases were managed without the need for biopsy—i.e., problem solving MRI in 5 cases and early follow-up CEM in 2 cases. All of these non-biopsy follow-up investigations were radiologically normal/benign, with a mean follow up period of 14 months and median 12 months. The remaining 66 (90.4% of the 73 recalled women) underwent either percutaneous biopsy, guided by US in 25/66 (37.9%), stereotaxis in 24/66 (36.4%) and, when the lesion could not be reliably identified by either US or MG, MRI (17/66; 25.8%). Table 3 Figs. 1, 2, 3.

**Table 3:** Recall rates and biopsy rates among recalled cases, according to breast density

	MD BIRADS				Total cohort
	A	B	C	D	
Recalled to assessment	1/69 (1.5%)	37/594 (6.2%)	31/437 (7.1%)	4/91 (4.4%)	73/1191 (6.1%)
Managed without biopsy	0	2	4	1	7
Biopsy performed	1/69 (1.5%)	35/594 (5.9%)	27/437 (6.2%)	3/91 (3.3%)	66/1191 (5.5%)
US-guided	1	17	7	0	25
Stereo guided	0	10	11	3	24
MRI-guided	0	8	9	0	17

Of the 73 recalled patients, 9 lesions were only seen on 2D/3DMG with no contrast enhancement (Table 4). Eight of these were small clusters of calcification, 1 was an architectural distortion and all 9 were FP on final pathology. A total of 23 lesions were identified using both 2D/3D MG and C + ; of these, 19/23 (83%) were TP.

**Table 4:** Results of cases recalled for further assessment, and the contribution of contrast

	Total	Benign*	Malignant			Diagnosis rate (of all women Surveilled)	PPV
			DCIS	Invasive cancer	Total malignant		
Identified without C +	32	14	4	14	18	1.51%	56.3%
2D/3D alone	9	9	0	0	0		
2D/3D + C +	23	5	4	14	18		
Identification directed by C + (C + directed)	41	24	5	12	17	1.43%	41.5%
Minimal signs 2D/3D and C +	14	3	2	9	11		
C + only	27	21	3	3	6		
Total	73	38	9	26	35	2.94%	47.9%

*On biopsy or follow-up imaging

Forty-one of the 66 biopsied recalls (62%) were unlikely to have been identified had contrast not been used (C + directed). Of these cases, 14/41 (34%) were considered to have ‘minimal signs’, in that with knowledge of enhancement, a lesion could be identified on 2DM/3DMG images alone, but was unlikely to have been identified without contrast assistance, while 27/41 were only identifiable with contrast (C + only) (Figs. 1, 2, 3). Of the 41 cases identified because of the use of contrast, 11 underwent US-, 7 stereo, 17 had MRI-guided biopsy and 6 required no biopsy with follow up as previously described.

17/41 were TP (12 invasive cancer and 5 DCIS). 11/14 “minimal signs” lesions were TP, as were 6/27 C + only lesions.

Assuming that ‘minimal signs’ cancers would not have been identified without contrast, the Cancer Detection Rate (CDR) was 15.1/1000 patients in the absence of Contrast and 29.4/1000 when it was used. The overall PPV was 47.9%, and was 41.5% in those recalled due to Contrast findings.

On histopathology, 6 of the 21 FP (benign) C + -only lesions were fibroadenomata, 8 were benign including fibrocystic change, sclerosing adenosis, duct ectasia, and fat necrosis and 1 contained scar tissue. Problem-solving MRI in 3 revealed a further single lesion as a leash of enhancing vessels, a fibroadenoma, and an area of duct ectasia. 3/21 lesions showed no enhancement on early follow up CEM or MRI.

Patients with malignant lesions found on CEM only were younger (*t*-test *p* = 0.049), with some indication of being less likely to have had DCIS and to have higher MD (Table 5). Additional cancer detection was not limited to those with high MD: 8 of 19 TPs in those with BIRADS B MD had “minimal signs” or were only found with contrast. The distribution, morphology and the size of the malignant lesions was similar between these two groups, as was the incidence of high-grade cancers and HER2 + ve or triple negative phenotype.

**Table 5:** Additional details of cases with malignant lesions (TP cases)

	Component of CEM leading to diagnosis			Total
	2D/3D & C +	C + -only	Test for difference	
Number of cases	18	17		35
Age—median (range) Mean	67 (46–77) 64.3	57 (35–76) 56.8	t-test p = 0.049	61 (35–77) 60.8
Time since Index Cancer (years) (median (range)) Average	3.1 (0.9—14.9) 4.9	3.1 (0.9—15.1) 4.9	t-test p = 0.923	3.1 (0.9—15.1) 4.9
Index Morphology DCIS Invasive cancer	8 (44%) 10 (66%)	5 (29%) 12 (71%)	Fisher’s exact p = 0.489	13 (37%) 22 (63%)
Mammographic density A B C D	1 (6%) 11 (61%) 6 (33%) 0 (0%)	0 (0%) 8 (47%) 8 (47%) 1 (6%)	Fisher’s exact p = 0.489	1 (3%) 19 (54%) 14 (40%) 1 (3%)
BPE (side of new disease) Minimal Mild Moderate Marked	1 (6%) 8 (44%) 9 (50% 0 (0%)	0 (0%) 6 (35%) 10 (59%) 1 (6%)	Fisher’s exact p = 0.790	1 (3%) 14 (40%) 19 (54%) 1 (3%)
Side Ipsilateral Contralateral Bilateral	11 (61%) 6 (33%) 1 (6%)	9 (53%) 8 (47%) 0 (0%)	Fisher’s exact p = 0.611	20 (57%) 14 (40%) 1 (3%)
Morphology— DCIS Invasive cancer	5 (28%) 13 (72%)	4 (24%) 13 (77%)	Fisher’s exact p = 1.000	9 (26%) 26 (74%)
Size (mm diameter) (median (range)) DCIS Invasive cancer	9 (2—20) 27 (8—85)	35 (20—60) 18 (4—141)	t-test p = 0.015 t-test p = 0.604	20 (2—60) 20 (4—141)
Grade of Inv ca 1 2 3 Unknown	1 (7%) 7 (50%) 5 (36%) 1 (7%)	2 (17%) 3 (25%) 7 (58%) 0 (0%)	Fisher’s exact p = 0.390	2 (14%) 10 (45%) 12 (55%) 1 (5%)
Node Positive	0	1		1
Phenotype of Inv cancer • ER + /her2- • ER + /her2 + • ER-/her2 + • TNBC	10 (77%) 0 (0%) 0 (0%) 3 (23%)	9 (75%) 0 (0%) 1 (8%) 2 (17%)	Fisher’s exact p = 1.000	19 (76%) 0 (0%) 1 (4%) 5 (25%)
Surgical treatment BCS TM None	9 (56%) 7 (44%) 2 (11%)	8 (47%) 8 (47%) 1 (6%)	Fisher’s exact p = 1.000	17 (49%) 15 (43%) 3^b^ (9%)

^a^1 malignant phyllodes with no pathological grade

^b^2 cases with metastatic disease and 1 with concurrent primary lung cancer at diagnosis

With the introduction of CEM, there was a steady decrease in supplemental whole breast US screening for surveillance. In the 12 months prior to the introduction of routine CEM in surveillance there were 2781 bilateral 2D/3DMG and 891 bilateral whole breast US in the same patients (32%). From July 2020 until June 2021, 2919 MMG (2074 were CEM, 845 without contrast) and 400 bilateral US were performed (14%), a 57% reduction in bilateral US (*p* < 0.0001).

## Discussion

This paper demonstrates that CEM as a single surveillance imaging test for those with a personal history of breast cancer or DCIS identified a significantly larger number of recurrences and new primary cancers than MG alone. Additional positive findings were found in all degrees of MD, and the pathology of these findings suggests they were clinically significant. A steady decrease in supplemental whole breast US screening for surveillance was noted over the time CEM was introduced with significant benefit to radiological services.

Local recurrence and new ipsi- or contralateral events comprise a substantial proportion of all breast cancer events in patients with PHBC. The frequency of these events is similar to that of mutation carriers undergoing surveillance. A meta-analysis by Lu *et. al.* of over 2000 patients demonstrated an improvement in survival when breast cancer recurrence was detected on radiological surveillance rather than symptoms [27]. Thus, surveillance imaging arguably should use a modality with the same sensitivity as that used for screening high risk groups.

CEM has been found to have a similar sensitivity profile to MRI and possibly higher specificity [19–21, 33]. When combined with 2D/3DMG, preferably in a single acquisition, it provides high quality supplemental screening in a single investigation. Due to the reported benefits of CEM, and limited capacity in our radiology department to support increasing routine surveillance US, we introduced CEM (in combo mode with 2D/3D) as a single standard surveillance test in late 2018. These results of a large series of cases of the first surveillance CEM support its use.

Cancers detected due to the inclusion of contrast are of particular interest, since early detection of these may improve outcomes. Alternatively, it is possible that additional disease would never have become clinically significant and using more sensitive imaging modalities will have the unintended consequence of increasing the rate of overdiagnosis. The pathology of the TP cases does not support this contention. The ratio of invasive cancer to DCIS was similar in those identified on 2D/3DMG and those that were C + directed (C + only or ‘minimal signs’). The proportion of invasive cancers that were grade 3 was numerically higher in the C + directed group, and the size of the cancers was similar in the two groups. A similar number of cancers were either HER2 positive or TNBC, and the only node positive case was in the C + directed group. Thus, while it is not possible to be certain, only a small proportion of C + directed cases identified had pathological features suggestive of possible overdiagnosis. These patterns need to be confirmed on a larger dataset.

MRI biopsy was required for over half the C + -only lesions. Breast services considering implementation of CEM need to take this into consideration. An important consideration is the need to confirm concordance between detected and biopsied lesions. We used marking clips and clip-check MG, sometimes with contrast to provide this. CEM guided biopsy is available in a small number of centres in Europe and the United States at the time of this report and is similar in performance to stereotactic biopsy. This should improve access, acceptability and also the cost effectiveness of the technique. However, since current CEM biopsy is limited to a single lesion per sitting, MR biopsy remains the most expeditious method to reliably biopsy more than one lesion.

Cases with minimal signs on 2D/3DMG and corresponding suspicious findings on contrast are considered likely to have been missed on MG alone, so the contrast plays an important role not only in detection, but also in directing attention to the “minimally” suspicious area and facilitating biopsy with conventional means.

Of the cases identified on 2D/3DMG alone (i.e. non-enhancing lesions), all 9 were false positive, suggesting that biopsy may potentially be avoided for such lesions where contrast is used. Importantly, over 40% of those that were either C + -only, or ‘minimal signs’ on 2D/3DMG were malignant.

MD is important, both as a risk factor for developing cancer and due to its masking effect. The majority of the TP biopsies identified on 2D/3DMG had BIRADS A or B MD and conversely, around half where the 2D/3DMG was normal or showing minimal signs had BIRADS B MD, suggesting the benefit of contrast is not confined to those with high MD. Some malignant lesions are associated with abnormal enhancement without morphological abnormalities on non-dense MG.

BPE is an issue with MRI and also with CEM, as it can potentially obscure significant findings. While high BPE on CEM is associated with high MD, most cases with high MD had low BPE. In this series, while 44% had BIRADS C or D MD, only 7% had moderate or marked BPE. This is lower than the expected rate of BPE, and may be due to the impact of adjuvant radiotherapy and/or systemic therapies. This is a significant advantage for the use of CEM in surveillance.

False positive recall is a harm of surveillance, and minimising the FP rate is important. Overall the addition of contrast did not significantly increase that proportion of FP results, despite increasing the absolute number of FPs compared to MG alone. The recall rate of 6.1% with CEM in this series was lower than a recall rate of 8.8% reported for surveillance MG in a large published series of women with PHBC [35]

Fibroadenomata and fat necrosis related to scars contribute to the FP rate amongst the C + only lesions. It is difficult to dismiss enhancement in a scar or bright mass enhancement in the prevalent round of surveillance CEM without biopsy. It is expected that the rate of these recalls will decrease over subsequent rounds with an established baseline.

Strengths of this study include its large number of surveilled women, with complete capture of consecutive cases. All patients were offered and most accepted. All findings on surveillance CEM were documented. This therefore is likely to represent the outcomes of a first surveillance round should CEM be adopted as a standard imaging modality.

Our data compares favourably with the literature, the Dutch DENSE trial investigating MR screening in the population of those with extremely dense breasts demonstrated a recall rate of 9.5%, PPV of 17.4% and FP rate of 79.8% compared to this study’s recall of 6.1%, PPV of 47.9% and FP rate of 52% [16]. Prior reports of CEM in surveillance or in screening of high risk patients have also reported benefit from CEM, and that around 50% of TPs were only identified with Contrast. The CDR in prior reports was around 15/1000 (Sung 2020, Gluskin 2019) [22, 23], which is lower than in this series. The recall rate in those series was similar to the current report, while the PPV was lower.

Limitations include limited cancer diagnoses, no data on the number of patients eligible for inclusion and the lack of subsequent round findings to capture interval cancers. This means it is not possible to determine the false negative rate, nor the sensitivity or specificity at this stage. The increased detection of TPs, and the identification of a number of lesions not evident on standard imaging suggest that the subsequent cancer rate—both interval and at subsequent screening rounds—will be lower and this will be the subject of future analyses. This is a heterogeneous cohort, with patients offered surveillance CEM at various times after their index cancer was treated. There was a variable use of contrast imaging—either CEM or MRI—at the time of diagnosis. Contrast imaging at initial cancer diagnosis was limited prior to 2018, so it is possible that some lesions found on surveillance CEM may have been present but occult at the time of diagnosis. This applies to both TP and FP cases.

## Conclusions

CEM as a single surveillance imaging test for those with a personal history of breast cancer or DCIS identified a significantly larger number of recurrences and new primary cancers than MG alone. Additional positive findings were found in all degrees of MD, and the pathology of these findings suggests they were generally clinically significant. Further study will assess the impact on subsequent diagnosis and interval cancer rates.

## Data Availability

The datasets generated during and/or analysed during the current study are not publicly available due to potential patient identifiable data but are available from the corresponding author on reasonable request.
